# HIV testing experiences and perceptions among Black transgender and nonbinary people in New Orleans, LA

**DOI:** 10.1080/15381501.2026.2617276

**Published:** 2026-02-09

**Authors:** Manuel A. Ocasio, M. Isabel Fernández, Sydney Clark, Karina Butani, Katherine P. Theall, Gary W. Harper

**Affiliations:** a Department of Pediatrics, Tulane University School of Medicine, New Orleans, LA, USA;; b Department of Social, Behavioral, and Population Sciences, Tulane University Celia Scott Weatherhead School of Public Health and Tropical Medicine, New Orleans, LA, USA;; c College of Osteopathic Medicine, Nova Southeastern University, Miami, FL, USA;; d Department of Epidemiology, Tulane University Celia Scott Weatherhead School of Public Health and Tropical Medicine, New Orleans, LA, USA;; e Department of Health Behavior and Health Education, School of Public Health, University of Michigan, Ann Arbor, MI, USA

**Keywords:** Transgender persons, HIV testing, health inequities, nonbinary

## Abstract

HIV testing is a critical strategy to addressing the disproportionate impact of HIV among Black transgender and nonbinary (TNB) individuals in the Southern U.S. We conducted 12 semi-structured qualitative interviews with Black TNB young adults in New Orleans to explore factors influencing HIV testing experiences and perceptions. We used a socioecological framework to guide analysis and identified themes across intrapersonal, interpersonal, organizational, and community levels. At the intrapersonal level, HIV anxiety shaped testing experiences and perceptions. Peer influence and patient-provider interactions emerged at the interpersonal level, and clinic operations at the organizational level. Community-level factors included stigma, medical mistrust, and confidentiality concerns. Participants provided actionable recommendations, emphasizing the need for instrumental support (e.g., transportation), hiring and training Black TNB clinic staff, and creating culturally resonant HIV testing programs. Community-informed strategies centering Black TNB voices are critical for designing effective HIV prevention programs that honor their identities and lived experiences.

## Introduction

Black transgender and nonbinary (TNB) individuals in the Southern U.S. are disproportionately affected by HIV. In 2022, 40 percent of all incident cases among transgender people were black transgender women, and 52% originated in the U.S. South (U.S. Department of Health and Human Services, 2024). HIV prevalence among transgender men is estimated to be 3.4% (Becasen et al., 2019), which is significantly higher than that in the general U.S. population (0.5%) (U.S. Department of Health and Human Services, 2024). Targeted efforts to curb the spread of HIV among Black TNB individuals in general and in the South, in particular, are essential to achieve a 90% reduction in new HIV diagnoses by 2030, as proposed in the U.S. Ending the HIV Epidemic initiative (EHE) (Fauci et al., 2019).

Among the strategic initiatives of the EHE are to diagnose people with HIV as early as possible and to prevent acquisition among people at risk for HIV (Fauci et al., 2019). Routine HIV testing has long been a critical gateway to accessing prevention services (e.g., PrEP and risk reduction counseling) for those at risk and linkage to care for those who are diagnosed with HIV. Considerable efforts have been dedicated to identifying barriers and facilitators of routine HIV testing and developing tailored interventions to promote routine testing (Frye et al., 2021; Rhodes et al., 2020). The socioecological model is a useful organizational framework for describing factors that impact the acceptance of routine HIV testing (Golden & Earp, 2012). The model posits that factors across multiple levels, ranging from intrapersonal characteristics to interpersonal, organizational, and community levels, interact and influence individual perceptions and behaviors. The model highlights the interconnectedness of various levels of influence as well as the reciprocal nature of the relationships between individuals and their environment. At the intrapersonal level, low perceived risk (Golub & Gamarel, 2013; Olakunde et al., 2022; Scheim & Travers, 2017) and anxiety about a possible positive result (Kobrak et al., 2022) are common reasons for not being tested. Interpersonal factors, such as negative interactions with providers (Costa et al., 2018; Harper et al., 2019; Logie et al., 2017; Scheim & Travers, 2017) and fear of status disclosure, play a role in HIV testing behaviors (Costa et al., 2018; Harper et al., 2019; Olakunde et al., 2022). At the organizational level, inadequate service delivery (Arrington-Sanders et al., 2020; Harper et al., 2019; Scheim & Travers, 2017) and a lack of awareness of services that could increase HIV testing (Frye et al., 2018; Harper et al., 2019), such as HIV self-testing, have been identified as barriers to HIV testing (Frye et al., 2015). Finally, housing instability (Andrinopoulos et al., 2015; Harper et al., 2019), lack of access to medical care (Bukowski et al., 2018), HIV-related stigma (Olakunde et al., 2022), and transphobia (Rosenberg et al., 2021) are significant community-level barriers associated with HIV testing. Although many studies included Black TNB participants, we did not find a single study that specifically focused on HIV testing experiences among Black TNB communities in the South, particularly in areas with high HIV burden.

New Orleans, an EHE priority jurisdiction, is currently ranked 6^th^ in the nation for HIV incidence with disproportionately higher rates among Black residents, who comprise 57% of the population (U.S. Census Bureau, 2023) A primary component of the EHE initiative is to allocate additional resources, expertise, and technology to develop and implement locally tailored EHE plans. To support the development of HIV testing programs that resonate with Black TNB in New Orleans, we conducted one-on-one interviews with young Black TNB adults to explore the following research questions:

1. What are the intrapersonal, interpersonal, organizational, and community factors that influence HIV testing experiences and perceptions among Black TNB in New Orleans?
2. What recommendations do Black TNB have for promoting routine HIV testing?

## Methods

### Study design

This study was grounded in an interpretivist paradigm, which assumes that meaning is subjective, socially constructed, and can be understood through individual perceptions and lived experience. We used an exploratory descriptive qualitative design drawing on principles of qualitative description and phenomenologically informed approaches (Hunter, McCallum, & Howes, 2019; Rendle et al., 2019). As such, our approach centered first-person accounts and the meanings participants ascribed to their HIV testing experiences to generate practice-relevant insights. We did not seek to identify the essential structures of HIV testing experiences which would have called for a formal phenomenological approach (van Manen, 2017). We used socioecological theory as a descriptive framework to summarize HIV testing experiences and perceptions and explored participant recommendations for promoting routine HIV testing.

### Participants and procedures

For this study, we combined Black TNB participants from two separate qualitative studies that used the same recruitment approaches and had identical questions on HIV testing experiences and perceptions and recommendations for promoting routine HIV testing. Across studies, we asked about best, worst, and usual HIV testing experiences and influences within each socioecological level. For the first study, we recruited Black transgender men and nonbinary participants, who received $50 for their time and effort. We recruited Black sexual minority men and transgender women for the second study and only included the transgender participants in the current study. Participants in the second study received $80 as compensation because the study consisted of both a qualitative interview and observation of social media use, thus requiring more time and effort.

The eligibility criteria were comparable between groups. To be eligible, participants had to: 1) be at least 18 and no more than 35 years of age; 2) identify as Black and/or African American; 3) have had condomless anal or vaginal/front hole sex in the past 12 months; 3) reside in New Orleans; 4) identify as transfeminine, transmasculine, nonbinary, gender non-conforming, two-spirit, a binary gender that differed from their sex assigned a birth (e.g., identify as man who was assigned female at birth), or other TNB identity not listed (e.g., genderfluid).

The participants were recruited using convenience sampling. Our recruitment approach included distributing flyers at community agencies (clinics and social services) that serve Black TNB young adults, posting on relevant social media outlets (Facebook groups, Instagram profiles), referrals, and peer-to-peer recruitment. Interested individuals completed a brief online screening to determine their eligibility. Respondents who screened eligible were routed to a separate screen and asked to provide their preferred contact information if they wanted to participate. A study team member provided additional information about the study and coordinated a suitable date and time to conduct the informed consent procedures and interviews. Prior to initiating the interview, the study team member reviewed the consent document with participants and, after addressing any questions or concerns, obtained written informed consent. The consent process and interviews were held virtually using the HIPAA-compliant Zoom software.

### Data collection

The first author drafted the initial version of the semi-structured interview guide with input from the study team and the coauthors GWH, KPT, and MIF. We also shared the interview guide with a youth advisory board comprised of local TNB and sexual minority young adults, the majority of whom identified as Black or African American. For Research Question 1, we created questions and prompts using the socioecological model to identify factors across multiple socioecological levels (intrapersonal, interpersonal, organizational, community) that influence routine HIV testing perceptions and behaviors. For example, at the organizational level we asked, “What things about the places where you got tested would make it easier?” and at the community level, “What sort of big picture things like policies, politics, religion, or common beliefs would make it easier for you to get tested?” The questions developed for Research Question 2 focused on recommendations for HIV testing programs for young Black TNB adults.

Between July 2021 and September 2023, we conducted 12 interviews that averaged approximately one hour each. The interviews were audio recorded and transcribed by the study team. The team consisted of four staff (including SC) and three faculty members (MAO, GWH, MIF) with experience in qualitative research methods, TNB health, HIV prevention, and varied backgrounds, identities, and experiences. The four team members (two staff, SC, MAO) who conducted the interviews were all non-white (75% Black) and all but MAO identified as part of the TNB community. The protocols used in this study were approved by the Tulane University Social and Behavioral Sciences Committee Institutional Review Board (2021–335-Online; 2021–492-Online).

### Data analysis

We used a general inductive approach for analysis and used Dedoose to organize data and facilitate coding and analysis (Dedoose, 2024; Thomas, 2006). For our process, we first developed an a priori codebook using the interview guide to create upper-level categories (e.g., socioecological levels) to organize codes. The first and second authors and the interviewers independently coded three transcripts using the a priori codebook, met to compare codes, resolve any discrepancies, and discuss incorporating additional codes to finalize the codebook. Subsequently, the first and second authors independently coded the remaining transcripts, met to compare codes, and resolve any discrepancies. We then developed lower-level subcategories (i.e., themes) based on these codes. Before finalizing our results, we shared our findings with our youth advisory board as a form of member checking to confirm our interpretations.

## Results

We first present participant characteristics and then the results organized by socio-ecological levels—intrapersonal, interpersonal, organizational, and community—with several themes present at each level. The themes we identified encompassed both positive and negative experiences and perceptions of HIV testing. We also describe themes for recommendations to promote HIV testing among Black TNB in New Orleans.

### Participant characteristics

The participants’ ages ranged from 19 to 34 years (median = 23 years). The sample is diverse in terms of gender identity. Twenty-five percent of participants were identified as transfeminine or transgender women, and half of the participants identified with more than one gender identity, such as nonbinary transmasculine or nonbinary transfeminine. The remaining participants identified as gender non-conforming, nonbinary, or man, respectively. All participants reported that they were tested for HIV at least once a year. Most participants integrated HIV testing into their routine healthcare; thus, the experiences they shared reflected encounters with clinics and providers that were not exclusively for HIV testing.

### Intrapersonal level

Intrapersonal level factors are characteristics such as thoughts or feelings that occur within the individual. At the intrapersonal (or internal) level, two main themes emerged: HIV anxiety and prioritizing sexual health.

#### HIV anxiety

Participants discussed how feelings of anxiety emerged, leading up to an HIV test. Anxiety was often linked to the fear of receiving positive HIV testing results in anticipation of testing. For example, a 24-year-old nonbinary interviewee said that “when you are unsure about whether you have HIV or any STI, there’s, like, a certain amount of anxiety—or at least for me—anxiety and doubt build[s] up in your mind.”

Another participant mentioned how they recounted experiences that may have exposed them to HIV:

I’m honestly thinking about all the people like I had sex with, their- like all of the experiences I’ve had like to even remotely think about a slip up, a possible slip up, in which you know, every time I think about you know I crossed on my I’s, dotted all my T’s. I forgot how you say it. I think I did it backwards.

(Gender non-conforming, 19)

In one rare instance, a participant described how their anxiety actually motivated them to undergo testing for HIV.

I definitely have a bit of anxiety. And so that motivates me to, you know, be proactive about things and be on top of what I need to be on top of ‘cause otherwise … I run through whatever scenarios, so I would say fear or anxiety around something is definitely a primary motivator.

(Nonbinary masculine, 19)

Thus, anxiety was a strong sentiment prior to testing and served as a motivating factor to seek testing.

#### Prioritizing sexual health

Participants cited HIV testing as a form of maintaining their sexual health and as critical to their overall health and well-being. One participant noted that HIV testing was a way to stay informed and maintain health:

Yes, I want to go test because [there is] nothing wrong with, like, checking your body and making sure you good. Like, and just knowing that, you know, like—it’s just good to be thorough with yourself and your health.

(Transgender woman, 19)

One participant iterated that the importance of HIV testing for their well-being supersedes the occasional lack of motivation to go to an HIV testing appointment:

There are days when, you know, I’m more on top of things than others, and sometimes I’ll wake up and have an appointment, and I really don’t want to go. I know I am doing it for my own well-being.

(Nonbinary masculine, 19)

By getting tested and exchanging their status with partners, study participants felt as though they were making informed decisions about their sexual health:

Uhm, it’s a way to like just know your status … it’s not a thing to stigmatize people or like, make it seem like they’re unsafe or unlovable, or not within community, but just a way to know like what’s going on with your body, so you can like take care of it in whatever ways you need to.

(Genderqueer, gender non-conforming, transmasculine, nonbinary, 30)

This theme underscores how HIV testing can be framed as an act of self-care and empowerment.

### Interpersonal level

Interpersonal level factors are related to the communication and relationships between individuals. At the interpersonal level, we identified two themes: peer influence and gender and racial dynamics in patient–provider interactions.

#### Peer influence

Peer influence impacted HIV testing experiences and perceptions in both positive and negative ways. For some, peer groups were influential in promoting sexual wellness and sparking discussions that prompted routine testing. For instance, a 30-year-old Genderqueer, gender non-conforming, transmasculine, nonbinary interviewee stated “A lot of people around me just to normalize [getting tested]. So, I think that it helps me feel comfortable with it. And like [my friends] are pretty open and share about [getting tested].” In contrast, other participants perceived their TNB peers as indifferent or non-engaged in promoting routine HIV testing. In particular, a 27-year-old nonbinary transmasculine participant reflected on this phenomenon in relation to “stealth” TNB, noting that his “stealth friends [are] like, ‘that doesn’t affect me,’ or they just think it doesn’t [matter for them].” Stealth refers to TNB people who choose to quietly, and seamlessly, integrate themselves into cis-gender and heteronormative spaces and do not acknowledge their TNB identity or history publicly (Transgender Language Primer, 2006n.d.). Thus, peer environments varied substantially. Affirming peers normalized testing while lack of discussion in other peer groups limited engagement.

#### Gender and racial dynamics in patient–provider interactions

Most participants integrated HIV testing into their routine health care and recognized that gender and race played a significant role in their interactions with clinic staff, ultimately shaping their perceptions of and experiences with testing. Their experiences were both positive and negative. It was common for participants to cite experiences of misgendering. For instance, a 27-year-old transfeminine participant noted that “It’s like someone will literally ask you your pronouns in like, you know, this sort of medical situation and then like misgender you.” Beyond being subject to misgendering, the same participant mentioned experiencing a general sense of dislike of transgender people from providers. The same participant said that some providers “act like they’ve never seen trans people before. And, like, sometimes it’s not the misgendering, but it’s like the fact that, like I can know that this person like does not like trans people … ” [Transfeminine, 27] On the other hand, another participant discussed a positive experience in which the provider’s tone instilled a sense of safety. A 22-year-old Genderqueer, gender non-conforming, genderfluid interviewee stated that “ … it did make me feel safer that, like, you know, there was no tone there. There wasn’t a tone from her that, like, inferred that she didn’t, like, you know … wasn’t comfortable with somebody like me.”

Generally, participants described their interactions as positive when staff and providers shared a Black and/or queer and/or TNB gender identity. One participant said “The nurse, was this Black, gay man, and he was just really cool, and I—I don’t know. Maybe I was more at ease, and I felt more comfortable, just like innately, from being in that space.” [Nonbinary, 24] Another participant expressed being more comfortable because their provider was a Black woman who listened to their concerns:

She actually listened. And I think that’s also a big thing is having people in the medical field who look like us and who also listened to us because like so many times, like having someone who looks like you makes you more comfortable.

(Nonbinary transfemme, 29)

Another participant elaborated that providers with similar lived experiences are less likely to discriminate:

Um, I don’t think it’s just about to have someone look like you. I think it helps you for them to have similar experiences as you … I think it’s definitely more comforting if you can go to a doctor who, like, may have a higher chance of not being discriminatory cause they’ve lived, like, as a Black person, or, like, as a queer trans person.

(Genderqueer, gender non-conforming, transmasculine, nonbinary, 30)

Some participants modified their speech and behavior when their identities differed from those of their providers. One participant described unconsciously “code switching” in order to be taken seriously when interacting with a white nurse:

I would say if I do have like—um I have a white nurse coming in—I may code switch and not speak to them the way I’m speaking to you. Yeah, I may do that whenever I do go into clinics or hospitals. I noticed I was doing this unconsciously, almost always bringing something related to school to the hospital—because of the fact that, like, people don’t take, like, people seriously in a hospital.

(Genderqueer, gender non-conforming, genderfluid, 22)

A Nonbinary, transgender man shared how their masculine presentation might be perceived at a women’s clinic while waiting for an HIV testing appointment:

I think that was the first time, where I realized that my demeanor was changed. I was like, I’m sitting in like a women’s clinic … And I’m sure everyone that’s like walking by– even if they weren’t, right, in my head, I’m like I’m sure everyone that’s walking by is like “what is this guy doing here”?

(Transmasculine nonbinary, 27)

This theme demonstrates how the intersecting dynamics of race, gender identity, and shared experience substantially shaped patient-provider interactions.

### Organizational level

Organizational factors are policies, practices, and norms within an organization. At the organizational level, clinic operations emerged as the main theme that influenced their HIV testing experiences and perceptions.

#### Clinic operations

When participants described testing for HIV, various aspects of clinic operations, such as efficiency and staff competence, significantly affected their testing experiences and perceptions. This theme emphasizes the functional aspects of clinic processes and services and not provider identities or quality of interpersonal communications. For some participants, testing for HIV was a straightforward process, and they appreciated that visits were quick and thorough:

It’s just basically a doctor or nurse giving me the rundown about everything in regards to like HIV and what-not, and then I just get a prick of blood taken off my finger. They do that really, like, fast test to test my blood and whatnot, and it’s nothing. Really, like, it’s just a couple minutes of waiting, and it’s done.

(Genderqueer, gender non-conforming, genderfluid, 22)

However, some participants noted that clinic hours were often limited and made it challenging to get tested. A 27-year-old transmasculine nonbinary interviewee stated that “I mean working a nine to five sometimes is annoying because, like, doctors close pretty early,” and limit access to testing for working patients.

A particularly favorable aspect of many participants’ testing experiences was the ability to communicate with providers and access results virtually through a patient portal. One participant stated that receiving HIV test results virtually was a preferred alternative to having a potentially negative conversation with clinic staff, based on their previous experiences:

I feel like I like it more now ‘cause everything like emailed to you or like put in the patient portal. But you know, I’ve definitely had a couple experiences where you know, some rude bitch was calling you up to, you know, get up in your business and be weird.

(Transfeminine, 27)

Most participants commented on the skills and knowledge of the clinic staff. For those who described positive experiences, the participants praised the staff who were skilled in drawing blood. For example, a 24-year-old Nonbinary patient described the staff at his testing clinic as “He was just like an angel, like the lightest touch. You barely felt the prick. The blood always flowed.” [Nonbinary, 24] On the other hand, some participants expressed frustration with inefficiencies they experienced during their visits.

You know I get there. I feel like. I talked to multiple people and it takes at least you talking to like, I’m gonna say 6 different people. Between the nurses and the doctors to talk to anyone who knows what they’re talking about, like then by the 6th person you’re like at the doctor who’s like the head of the department, but like they’re super busy.

(Transfeminine, 27)

Another participant described a particularly troubling experience in which the clinic failed to conduct a confirmatory HIV test after initially testing positive:

And then they did an HIV test and came back positive. But they did not go confirm the test. You know once you test positive, you supposed to confirm the test … I tested at my primary care physician. And she was like, ‘well, it’s negative.’ So, they [when] told me it was positive I was kinda like: the fuck? … So, my primary … she did the first test [again and] it came back negative, but [then] she did 2 confirmative tests and they were negative also. So, I was like damn. [The first place] had me fucked up.

(Transgender woman, 22)

These accounts portray how various aspects of clinic processes, including navigating clinic visits and obtaining results play a critical role in shaping perceptions in HIV testing services.

### Community level

Community-level factors include beliefs, values, norms, and resources within a given community or social group. Five themes were identified at the community level: confidentiality concerns, medical mistrust, stigma, and access.

#### Confidentiality concerns

Because the local TNB community is close-knit, the study participants were deeply attuned to how their health-related behaviors, including HIV testing practices, would possibly be disclosed among community members. Specifically, one participant described changing testing locations because he knew too many people and was concerned that his status would be shared in the community if he tested positive:

We used to go to like [Clinic] but we started knowing a lot of people there so we stopped going there … in New Orleans [the clinic workers are] a lot of gay people, you know … I just feel like if I’m [HIV] positive … you don’t have to tell people. Some places that you go they really run they mouth.

(Man, 34)

Because of concerns with confidentiality, one participant mentioned occasionally buying home tests:

So say, me and my boyfriend or me and one of my friends want to do like you know testing at home and they don’t want everybody to know their results or something like that then we can just do it at home and they’ll be very confidential.

(Transgender woman, 22)

Overall, these findings highlight confidentiality as a central concern influencing HIV testing decisions, particularly in close-knit communities where fear of disclosure may hinder clinic testing.

#### Medical mistrust

Participants spoke about their mistrust of the medical system and how this influenced their HIV testing experiences and behaviors. According to one participant, mistrust tended to manifest biases from the providers themselves, alongside other clinic staff.

I think yeah, institutionalized racism in the healthcare system—[it’s] just ongoing and unaddressed … So, it still shows up in biases from like doctors and providers, even when they think they’re showing up how they should; they’re still showing up with biases.

(Genderqueer, gender non-conforming, transmasculine, nonbinary, 30)

For one participant, medical mistrust made conversations about HIV prevention including HIV testing with their Black TNB friends challenging:

Of course, there’s some friends who are … who are like … I don’t wanna say conspiracy theorists, but just distrustful, right, of medicine. And that comes into play a lot. So, it kind of derails conversations when we do get on topics like these [HIV testing].

(Nonbinary transmasculine, 27)

One participant noted a particularly negative HIV testing experience in which medical students were present during her clinic visit and made her feel like an experiment:

I’ll be used as like a fucking like experiment like, you know. In front of people like they’ll have like a teachable moment … I don’t know if it’s like in the forms that you sign that you consent to this, but it’ll be like a group of people watching and like learning. But it’s like I don’t know when I would have consented to this.

(Transfeminine, 27)

Together, these accounts highlight how medical mistrust manifested in provider biases, peer conversations about HIV testing, and dehumanizing clinic encounters.

#### Stigma

Stigma emerged as a prominent community-level theme, specifically relating to inherent assumptions about TNB people and their sexual behaviors that impact their HIV testing experiences and perceptions. One participant described feeling “embarrassed” when going to a provider for testing and PrEP medication because they feel like they are upholding stereotypes that present queer people as “overly sexual”:

I feel like I’ve been embodying this stereotype a little bit in that way. Like, in like, ‘Hey, I’m having all the sex. I’m worried about all these different things that I could get from having all this sex.’ I feel like I’m like embodying what they perceive queer people to be like … I will say … I’ll say this: every time I’ve gone to the doctor’s office, for the past year, I’ve had some kind of question in relation to my sexuality or my sexual activity and it feels a little weird.

(Nonbinary masculine, 19)

Similarly, a 22-year-old transgender woman participant noted that because of the sexual and gender identities of her partners, providers assumed that she had HIV. She stated, “I don’t have HIV and just cause they feel like I mess with a trans woman and I mess with gay men that I have it, but I really don’t.” (Transgender woman, 22). Another transgender woman participant (age 27) mentioned that there is an “assumption that all trans people have HIV and should be treated for it. Immediately, without their consent or like you know, we’ll be told.”

One participant acknowledged that stigma and discrimination were an inherent part of their daily lives, but these experiences did not deter them from being tested:

I guess [it is] the sense of the judgment [that stops some people], you know. Which—you know, we we’re gonna be judged every day, you know. They judge us about being gay; they judge about being trans; they judge us about being Black. [Laughs.] You know? So, hey, you know, it’s just something that we’ve learned to live with. You know you know I’m not gonna stop it from you know me.

(Gender non-conforming, 19)

Stigma, which is pervasive in TNB people due to assumptions about gender identity and sexual behavior, is deeply embedded in HIV testing experiences, even among those who remain committed to routine testing.

#### Access

Most participants noted that access was a key determinant of HIV testing in the Black TNB community. The participants described difficulties in accessing reliable transportation to the testing locations. A 22-year-old transgender woman stated, “I forgot to tell transportation, or I do not have the money to get on the bus. Then I be like, ‘Fuck; how am I supposed to get to my appointment? One participant in college described how, unlike many other Black TNB, they were able to get tested regularly because it was readily available on campus:

I got tested regularly in college, but I am also talking to folks like go to college right or didn’t have like the access to kind of get to college. So, they … weren’t really exposed to like this place where you could get tested.

(Transmasculine nonbinary, 27)

Structural barriers, including lack of access to care, unreliable transportation, and financial instability, minimize HIV testing opportunities among Black TNB people.

### Recommendations

In addition to discussing HIV testing experiences and perceptions, we also asked participants for their recommendations to promote HIV testing among Black TNB in New Orleans. Recommendations were consistent across participants and were organized into three themes: instrumental support, training and hiring clinic staff, and Black TNB-specific programming.

#### Instrumental support

Participants were aware of the pervasive impact of structural barriers on routine HIV testing among the Black TNB community in New Orleans. One participant noted, “Like make things not a cash money barrier for people because I just feel like people are suffering from like overwork and poverty and like lack of access.” [Genderqueer, gender non-conforming, transmasculine, nonbinary, 30] As such, participants recommended various ways to alleviate economic constraints, such as providing food and transportation at testing sites. A common recommendation was to directly incentivize Black TNB people for testing. One participant noted that the need for money overshadowed other approaches, such as specialized programs and educational initiatives to increase HIV testing:

Well like, fuck a program, give these n***** money. That’s what I’m saying, like, that’s what people really need. Everyone wants to make us some cute program or something … A concert. That’s cool. Educational shit. Blackboard. People need money is what they need. They need money and access to resources to do what they want.

(Nonbinary transfemme, 29)

According to participants, addressing basic needs, particularly in the form of economic assistance, is critical to promoting HIV testing among Black TNB in New Orleans.

#### Training and hiring clinic staff

Participants recognized the critical need to improve interactions with clinic staff and emphasized the need for more Black TNB representation and tailored training to improve HIV-testing experiences. A 24-year-old nonbinary participant recommended addressing bad experiences with providers by “just hiring more Black queer people to like be doctors and nurses, cause like I’m just hearing things from other people who are having negative experiences that they experiences are negative because of who they have testing them”. Some participants provided specific suggestions regarding the training content. For example, one participant mentioned the need for providers to better approach sexual health conversations, stating that “the provider can, you know, play a role in making that conversation easier and starting that conversation. So having questions that open up to their patients” (Nonbinary masculine, 19). When asked to provide recommendations for providers and community-based organizations to improve how they provide sexual health services such as HIV testing to Black TNB youth in New Orleans, one participant pointed out the need for training among providers to avoid unnecessarily associating gender with medical conditions:

Like you know, your gender literally has nothing to do with Uh, what is being affected like say if you have like a cold or something or like you know if you have like literally back pain and like you know people are assigning like gender to it, so I feel like gender neutrality on healthcare would be great.

(Transfeminine, 27)

Participants emphasized the importance of clinics in hiring staff that who are part of their communities and providing specialized training on quality TNB patient care.

#### Black TNB specific programming

Participants stated that there was a lack of HIV testing events geared toward Black TNB in New Orleans and suggested several events, such as themed movie nights, queer field days, and concert series featuring local artists that could be coupled with HIV education and testing. One participant emphasized that these events should be “for Black queer people, by Black queer people … a space to exist, to commune, to share thoughts ideas, maybe even a meal” (Nonbinary transfemme, 29). Notably, participants identified that HIV testing events may not be one-size-fits-all and are often not sensitive to the unique experiences of some Black TNB people. One participant described the challenges of affirming Black stealth TNB people in healthcare:

Because in a healthcare setting you don’t really have the option of being stealth. So, are they less inclined [to get tested] because, like in that setting like … they have to out themselves. And how do we like create a space for folks who are comfortable in their stealthness to then get tested, right? How do we affirm their gender?

(Transmasculine nonbinary, 27)

Another participant pointed out that transgender men are often not aware that HIV testing events are also for them: “ … it’s not just trans women, it’s men too. I need them to know it’s men too.” (Man, 34)

Participants stated that HIV testing events should be intentionally tailored to the needs and interests of the Black TNB community and thoughtfully designed to accommodate subcommunities with distinct needs, such as TNB who are stealth.

## Discussion

In this study, we explored the socioecological factors that influence HIV testing experiences and perceptions and garnered recommendations for improving HIV testing engagement among Black TNB in New Orleans. It is interesting to note that most participants were tested regularly and incorporated HIV testing as part of their routine primary and gynecological care. Thus, at times, it was difficult to separate their HIV testing experiences from their overall clinic visits, and the narratives they shared should be viewed through this lens. Many of our findings mirror previous work, such as experiencing anxiety and fear in anticipation of testing (Scheim & Travers, 2017) and reporting both negative and positive experiences with clinic operations (Harper et al., 2019).

At the interpersonal level, peers played a particularly important role in shaping HIV testing experiences and perceptions. Peers who share similar minoritized identities and experiences are important resources for learning about sexual health and for encouraging HIV testing. HIV-related peer support is associated with increased testing intentions and self-efficacy among Black transgender women (Lelutiu-Weinberger et al., 2020). However, not all peer groups are supportive. One participant noted having a friend group of “stealth” TNB who did not openly discuss sexual health. It is possible that among some Black TNB who are “stealth,” the stigma surrounding HIV and gender identity may be internalized and deters Black TNB peer groups from discussing and addressing sexual health needs including routine HIV testing. A qualitative study examining barriers to HIV prevention engagement among TNB women noted how being “stealth” was critical for personal safety and potentially caused by shame (De Santis et al., 2023). Interventions that promote peer support significantly improve HIV prevention and care engagement among transgender women and should be explored for Black transgender men and other TNB at risk for HIV.

Having healthcare providers with racial and/or cultural backgrounds similar to the participants played an important role in their HIV testing experiences. Many participants reported positive experiences with providers and staff because they shared a Black and/or TNB identity and described negative interactions, specifically with white providers. Furthermore, participants also recommended hiring Black TNB providers and staff as an approach to improve clinical experiences, thereby increasing HIV testing. Prior research has shown that TNB patients have a preference for shared identity with their providers, and discordance in clinician-patient TNB identity has been associated with care avoidance (Liu et al., 2024). In fact, racial concordance is associated with higher levels of perceived patient satisfaction, comfortability, safety, quality of care, and trust in their providers. Moore et al. (2023) found that racially discordant providers were able to compensate for racial differences by exhibiting cultural humility and increasing awareness of racial disparities, which could be addressed through formal training. Further research should explore how racial and gender identity concordance and interactions between Black TNB patients and providers influence HIV prevention engagement and other healthcare experiences.

Many participants expressed concerns that their testing behaviors and results could be disclosed to others in the Black TNB community. This concern directly contradicts the reported positive experiences with Black TNB clinic staff and recommendations for Black TNB representation in clinics. Thus, hiring Black TNB staff and providers alone is insufficient to sustain HIV prevention engagement and should also be accompanied by enhancing clinic-wide privacy infrastructure. We suggest pairing workforce diversification efforts with implementing rigorous structural safeguards, such as standardized confidentiality training and robust accountability mechanisms that could mitigate these concerns. Furthermore, reconfiguring the location and workflow for HIV testing within clinics, such as implementing discrete check-in procedures and integrating waiting areas that preserve anonymity, may help mitigate concerns about disclosure (Logie et al., 2017). One participant chose home-based testing as a discreet alternative, which has been shown to be highly acceptable for transgender women (Lippman et al., 2016; Wood et al., 2021). Some participants opted to visit clinics in other locations to avoid potential disclosures. Given that participants cited a lack of transportation as a barrier to testing, this may not be an easily accessible option for many Black TNB, and interventions should focus on directly addressing disclosure concerns at clinics.

At the community level, medical mistrust emerged as a theme that impacts HIV testing experiences and perceptions. Although increased medical mistrust is associated with decreased healthcare utilization (LaVeist et al., 2009), it is also counterintuitively associated with increased HIV testing among Black sexual minority men (Simon et al., 2024) and Black Americans more broadly (Bogart et al., 2019). It is possible that people prioritize their self-care despite having mistrust or, because of mistrust, repeat testing to confirm the results (Simon et al. 2024). This mistrust may be well-founded. One participant shared an experience of receiving a false positive result without confirmatory testing and later sought additional testing, which confirmed a negative result. We could not identify any studies exploring the relationship between medical mistrust and HIV testing among Black TNB specifically, indicating the need for additional research in these communities.

In addition to describing their HIV testing experiences and perceptions, participants provided recommendations for increasing routine HIV testing among Black TNB in New Orleans. There was an overwhelming recognition that many Black TNB need instrumental support (e.g., money, food, and transportation) to mitigate the impact of structural barriers that impede access to testing and subsequently increase HIV testing engagement (Poteat et al., 2017). In addition to addressing structural needs, participants emphasize the importance of HIV testing programs that are designed for Black TNB communities. Consistent with recommendations, effective programming should integrate instrumental support with community-based events and gatherings to foster trust and reduce barriers (Poteat et al., 2022). This points to the importance of creating systemwide changes that facilitate more equitable distribution of resources and opportunities to help Black TNB address their basic needs.

In a systematic review of 23 interventions for improving engagement in HIV prevention and care among transgender people in the U.S. (Crepaz et al., 2025), 94% were designed for transgender women of color, 70% directly addressed structural barriers, and 20 showed improvement in at least one HIV-related outcome. Despite these promising developments, this review also reveals several gaps. Only one intervention focused on HIV testing, and there was a dearth of interventions for transgender men, which echoes participant recommendations for HIV-testing programs that are explicitly inclusive of transgender men.

Our study has several limitations. We used various recruitment methods to enroll Black TNB young adults with diverse gender identities in New Orleans. Because all participants in our sample were frequently tested for HIV (i.e., testing at least once a year), our results may not accurately reflect experiences and perceptions among Black TNB who infrequently or never test for HIV, who may be qualitatively different. Nonetheless, participants provided insight into the perspectives of their Black TNB friends, which included Black TNB who did not test frequently. Furthermore, our study was also limited to New Orleans, a city with a distinct sociocultural context and HIV service landscape, meaning that some findings may not be transferable to other geographic areas. All interviews were conducted on Zoom, which may have influenced rapport-building, depth of discussion, and ability to observe nonverbal cues (Schlegel et al., 2021). Additionally, we combined participants from two separate studies; although the interview questions and analyses were consistent, differences in study context and incentives may have shaped participant responses. Lastly, despite having a relatively small sample size, we achieved data saturation which can be achieved with as few as nine participants in studies with relatively homogenous populations (e.g., Black TNB in New Orleans) (Hennink & Kaiser, 2022).

## Conclusion

HIV disproportionately affects Black TNB people in the South, and HIV testing is a key intervention to reduce this burden. The socioecological framework facilitated the exploration of complex themes at different levels—ranging from the intra- and interpersonal levels to organizational systems and community factors—that affect HIV testing perceptions and experiences. Additionally, we identified actionable recommendations to increase routine HIV testing and opportunities for targeted interventions among Black TNB people in New Orleans to achieve the goals of the EHE initiative. Focused efforts are needed to create healthcare settings that affirm Black TNB identities and experiences and address structural barriers (Poteat et al., 2019). Our study also highlighted the need for HIV prevention programs to ensure privacy and confidentiality and consider the unique experiences and perceptions of often-overlooked Black TNB subpopulations, specifically Black transgender men and “stealth” TNB people. By using approaches directly informed by Black TNB people, we can design and implement HIV prevention research and programs that respect their identities and lived experiences, while addressing the structural inequities they face.
